# Pre-attentive Mismatch Response and Involuntary Attention Switching to a Deviance in an Earlier-Than-Usual Auditory Stimulus: An ERP Study

**DOI:** 10.3389/fnhum.2019.00058

**Published:** 2019-03-06

**Authors:** Pekcan Ungan, Hakan Karsilar, Suha Yagcioglu

**Affiliations:** ^1^Department of Biophysics, School of Medicine, Koc University, Istanbul, Turkey; ^2^Department of Psychology, Özyegin University, Istanbul, Turkey; ^3^Department of Biophysics, Faculty of Medicine, Hacettepe University, Ankara, Turkey

**Keywords:** MMN, P3a, regular stimulation, inter-onset interval, pitch, attention switching, additivity

## Abstract

An acoustic stimulus elicits an electroencephalographic response called auditory event-related potential (ERP). When some members of a stream of standard auditory stimuli are replaced randomly by a deviant stimulus and this stream is presented to a subject who ignores the stimuli, two different ERPs to deviant and standard stimuli are recorded. If the ERP to standard stimuli is subtracted from the ERP to deviant stimuli, the difference potential (DP) waveform typically exhibits a series of negative-positive-negative deflections called mismatch negativity (MMN), P3a, and reorienting negativity (RON), which are associated with pre-attentive change detection, involuntary attention switching, and reorienting of attention, respectively. The aim of the present study was to investigate how these pre-attentive processes are affected if the change occurs earlier than its usual timing implied by isochronous standard stimuli. In the MMN paradigm employed, 15% of the standards were randomly replaced by deviant stimuli which differed either in their pitch, their earlier onset time, or in both. Event-related responses to these three deviants [timely pitch change (R_TP_), earlier onset (R_EO_), earlier pitch change (R_EP_)] and to standards (R_S_) were recorded from 10 reading subjects. To maintain identical stimulation histories for the responses subtracted from each other, “deviant-standard” difference potentials (DP) for “timely” and “early” pitch deviances were derived as follows: DP_TP_ = R_TP_ − R_S_ and DP_EP_ = R_EP_ − R_EO_. Interestingly, the MMN components of the DPs to timely and early pitch deviances had similar amplitudes, indicating that regularity of stimulus timing does not provide any benefit for the pre-attentive auditory change detection mechanism. However, different scalp current density (SCD) dynamics of the MMN/P3a complexes, elicited by timely and early pitch deviances, suggested that an auditory change in a stimulus occurring earlier-than-usual initiates a faster and more effective call-for-attention and causes stronger attention switching than a timely change. SCD results also indicated that the temporal, frontal, and parietal MMN components are simultaneously present rather than emerging sequentially in time, supporting the MMN models based on parallel deviance processing in the respective cortices. Similarity of the RONs to timely and early pitch deviances indicated that reorienting of attention is of the same strength in two cases.

## Introduction

An acoustic stimulus elicits in the brain an electrical response called auditory event-related potential (ERP), which can be recorded non-invasively from the scalp by means of electroencephalography. When some of the members of a stream of standard auditory stimuli are replaced randomly by a deviant stimulus and this stream is presented to a subject who does not pay attention to the stimuli, two ERPs are elicited by deviant and standard stimuli, respectively. If the ERP to standard stimulus is subtracted from the ERP to deviant stimulus, the difference potential (DP) waveform typically exhibits a negative wave which peaks at about 120–200 ms from the onset of deviance and a positive wave which peaks in a latency range of about 200–300 ms. The former of these waves is called mismatch negativity (MMN) and, because of its automatic elicitation, even without attention to stimuli, it is associated with the brain’s involuntary and pre-attentive change detection and used as an index for this process as well as the initiation of attention switch towards the changes (Näätänen, 1992).

According to source-localization studies (Giard et al., 1995; Levänen et al., 1996), the MMN responses to changes in different aspects of an otherwise regular stimulus are generated at slightly different brain areas, supporting their independence. The subsequent positive wave called P3a, which is recorded with maximal amplitudes at fronto-central electrodes like the MMN, is not quite understood in the context of a typical MMN experiment, where the subject is required to ignore the stimuli. This is because the P3a is typically elicited in response to a distractor when the subject is actively attending to a target stimulus (Näätänen, 1990; Polich and Criado, 2006). However, there seems to be a consensus in the literature that the P3a reflects involuntary shifts of attention as an electrophysiological correlate of the orienting response, and is associated with the actual attention switch (Escera et al., 1998; Schröger and Wolff, 1998) that is believed to be triggered by a call-for-attention mechanism linked to the frontal component of the MMN. Following the P3a, a later negative activity around 400 ms may be seen in the ERPs to deviant stimuli. This component is centered on fronto-central electrodes and referred to as reorienting negativity (RON; Schröger and Wolff, 1998). It has been shown to provide a neurophysiological index of reorienting of attention, which has been switched towards the preceding deviant stimuli (Berti et al., 2004). Although this series of negative-positive-negative ERP components called MMN, P3a, and RON are associated, respectively, with pre-attentive change detection, involuntary attention switching, and reorienting of attention, there are studies questioning the hypothesis that they form a strongly coupled chain reflecting the sequential stages of auditory change detection and distraction (Horváth et al., 2008b).

The aim of the present study is to investigate how the auditory pre-attentive processing of stimulus-change and the subsequent switching of attention towards the deviant stimulus are affected from occasional shortening of the otherwise regular interstimulus interval. In other words, we aim to see if an auditory change occurring earlier than its standard timing is processed differently to the same change occurring at its usual time. We use the above-mentioned MMN, P3a, and RON components of the electrical brain responses for this purpose.

It was shown that a regular temporal auditory pattern can entrain expectations or attention involuntarily (Jones et al., 2002), suggesting an automatic, stimulus-driven entrainment of attention or involuntary temporal orienting. In the mentioned study, the authors examined a form of stimulus-driven attending that involves temporal expectancies influenced by stimulus rhythm, and observed that listeners were most accurate when judging the pitch of rhythmically expected tones and least accurate with highly unexpected ones. Based on this observation, which is in contrast theoretically with the view that people attend to pitch independently of time (for a review, see Krumhansl, 2000), they proposed a model involving an attending oscillator driven by a regular stimulus rhythm with fixed inter-onset intervals (IOIs) between rhythmic tones.

Inspired by the above-mentioned finding indicating stimulus-driven entrainment of directed attention, we investigate in the first place whether the central mechanism responsible for the function of pre-attentive change detection is similarly entrained by rhythmic stimuli. Studying this issue should also be interesting if the observations of Lange (2009) are considered. It is reported in that study that both temporal and pitch expectations modulate the N1 wave of the auditory ERP, indicating modulation of the early perceptual auditory processing by expectations. We focus, however, on the levels preceding those that have previously been addressed in studies linking regular temporal auditory patterns to expectations or involuntary attention (Jones et al., 2002). More specifically, we will try to find out whether or not the MMN, which represents the auditory change detection process below the attentive levels, is synchronized with or modulated/entrained by the rhythm of an isochronous auditory stimulation in such a way that its sensitivity or responsiveness will be maximized at the most probable times of stimulus occurrence and minimized when this probability is relatively low. Presence of such an entrainment would be interesting because it may indicate a pre-attentive mechanism facilitating involuntary temporal extrapolations and expectancies, which should be important for adaptation of the organism subconsciously to its auditory environment and, for efficient use of limited resources in the brain allocated for attention.

A modulation of this type may be associated with a detection advantage for a change in a rhythmic stimulus over a change in an irregularly occurring stimulus in the sense that all the deviants will occur, in the case of rhythmic stimulation, when the mismatch detection mechanism is maximally sensitive; whereas for irregular stimulation there will be no such special time periods in which its sensitivity would particularly be enhanced. Based on the established view that pre-attentive detection of a change in stimulus is indexed by MMN (Näätänen et al., 1993, 2012; Kujala and Näätänen, 2003), one may expect recording larger amplitude-MMNs with rhythmic stimuli than with irregularly timed stimuli if the above mentioned change detection advantage for regularly timed stimuli is valid. Indeed, there are studies reporting larger amplitudes for MMNs to isochronous stimuli than those to stimuli with randomized inter-stimulus intervals (Takegata and Morotomi, 1999). However, there are others either reporting (Schwartze et al., 2011) or assuming (Levänen et al., 1993; Paavilainen et al., 2001) equality of the MMN magnitudes recorded in the two cases.

Schwartze et al. (2011) found no significant difference between the MMNs they recorded with isochronous and random stimulus sequences. Indeed, such a finding indicates that temporal regularity of stimuli does not provide an advantage for the automatic mismatch detection mechanism. However, it is an overall finding concerning the whole stimulus sequences and does not provide information as to whether the responsiveness of the mechanism remains stable during an isochronous sequence and does not display changes in correlation with the timing of the deviance, with respect to the onsets of standards when they are regular. For instance, a possible drop in the responsiveness that would be specific to earlier deviances might have been masked in their random sequence experiments because they evaluated the MMNs, not only to deviances which were distinctly earlier than the timing cued by the average IOI, but also to those which were only slightly earlier or even later than that. In this scenario, where the participants are assumed to be smart enough to readily discover the average IOI, the hypothesized stimulus-entrained MMN mechanism will be affected only by deviances which are distinctly earlier than the average timing; and, even if its sensitivity drops for these deviances, this may fail to result in a significant difference between the amplitudes of the MMNs recorded with regular and irregular IOIs. An alternative scenario would be as follows: when the sequence is irregular, the mismatch detection mechanism will have no hint for the time of occurrence of the next stimulus. Sensitivity of the mechanism would therefore remain high (constantly active) throughout the whole sequence including the time periods in between the onsets of successive stimuli, and a possible advantage of temporal regularity might have thus been masked in the mentioned study. In either one or in both ways mentioned above, the results of that study might have indicated no significant MMN advantage for regular IOIs even if the mismatch detection mechanism worked in the hypothesized manner.

There are two other studies in which the event-related magnetic fields (Levänen et al., 1993) or electrical potentials (Paavilainen et al., 2001) were recorded in response to pitch-deviant stimuli, occurring at instances cued by a standard stimulus onset asynchrony (SOA) and at instances earlier than that time. In both studies, where the main interest was testing the hypothesis of additivity of the MMN or its magnetic counterpart MMNm elicited simultaneously by more than one deviant, the MMN elicited by an earlier pitch deviance was compared with its predicted waveform modeled by summing the corresponding single-deviant MMNs. In this prediction, equivalence of the MMNs (or MMFs) elicited by timely pitch deviances with those to be elicited by early pitch-alone deviances were assumed. This assumption is, in fact, the hypothesis tested in the present study.

However, in a recent study by Althen et al. (2016), where the main concern was testing single and double deviance-related modulations of the middle latency response, the cortical MMN to double deviant stimuli was shown to be smaller than the modeled double deviant MMN, composed of the sum of the single deviant MMNs. The authors explain this observation favoring sub-additivity of cortical MMNs, which is in contrast with the results of earlier studies indicating their additivity, by the fact that both deviances in the double deviant used in their study were in the physical features of stimuli, whereas in the previous studies one member of the double deviance was in the temporal features of stimuli.

The MMN studies above are reviewed and discussed because of their relevance to the present study. However, they address different research issues to the one treated here. Therefore, although they are relevant, their findings do not provide a direct and accurate answer to our question as to whether isochronous stimulation has a modulating effect on pre-attentive change detection. Because such an effect would cause a difference between the deviance-responses to stimulus-changes occurring at and earlier than the standard stimulus onset time, a more direct experimental design for comparing these two kinds of responses is employed here as explained in “Materials and Methods” section.

The second aim of the present study is to investigate the temporal relationship among different components of the MMN when the occasional auditory change occurs earlier than the time suggested by the regular standard stimuli. We use scalp current density (SCD) mapping which provides a descriptive and qualitative spatial analysis of the ERP components at various latencies, refining interpretation of their topographies and thus improving the understanding of the underlying neurophysiological processes according to Giard et al. (2014). The MMN has a multi-component structure and is recorded with maximal amplitudes at fronto-central and central scalp electrodes (with a mastoid reference), exhibiting the strongest current source densities in temporal and frontal areas of topographic scalp maps (Sams et al., 1985). The main cortical sources of MMN are localized within the superior temporal plane (Scherg et al., 1989; Alho et al., 1998). In addition to this bilateral supratemporal component, which is associated with auditory feature analysis and deviance detection, there are studies suggesting a frontal component (for a review, see Garrido et al., 2009), which is associated with an attentional-call type of process (Öhman, 1979), causing involuntary switching of attention to changes in the auditory environment (e.g., Giard et al., 1990; Deouell et al., 1998; Rinne et al., 2000). In the study of Rinne et al. (2000), it is further reported that the frontal component of the MMN is activated slightly (8 ms) later than the temporal one. Opitz et al. (2002) also describe such a chronological distinction between the two MMN components based on their fMRI study. They report that the strength of the temporal activation is correlated with the amplitude of the change-related ERP at latencies around 110 ms, while the frontal activation is correlated with the change-related ERP at around 150 ms. These findings, suggesting a serial type of mismatch processing, are challenged, however, by the results of a SCD mapping study (Yago et al., 2001) reporting earlier significant activity in right frontal areas than in temporal areas. Also, Shalgi and Deouell (2007) question a canonical model in which the frontal MMN generator is contingent upon the activation of the temporal MMN generator, and suggest a parallel distributed processing type of network which would explain the discrepant results in the literature regarding the time lag between the temporal and frontal components of the MMN.

Besides the temporal and frontal components of the MMN, a parietal-lobe contribution to auditory change detection was suggested by several electrophysiological (Levänen et al., 1996; Kasai et al., 1999) and fMRI (Schall et al., 2003; Molholm et al., 2005) studies. In Levänen et al. (1996), parietal source locations of the fields evoked by various deviants were found to be not significantly different from each other, probably reflecting the activity of more global and nonspecific change detectors. And, based on the SCD maps plotted for different post-deviant latencies, Kasai et al. (1999) reported that the parietal MMN source was activated after the temporal and frontal sources. We investigate the temporal relationship among these different components of the MMN when the occasional auditory change occurs earlier than the time hinted, by regular timing of the standard stimuli. Temporal dynamics of the SCD maps of the responses to pitch-changes occurring at and earlier than the standard stimulus onset time are used for this purpose.

We also study the P3a and RON responses in the “deviant-standard” DP to a change in an auditory stimulus occurring earlier than its usual timing. Such an early change is a double deviance consisting of a pitch change coupled with shortening of the IOI. Althen et al. (2016) reports that the P3a response to double deviants is smaller in amplitude than the sum of the P3a responses to the constituent single deviants; that is, the P3a behaves sub-additively. Paavilainen et al. (2001), on the other hand, report that the P3a to the double deviance of stimulus frequency and inter-stimulus interval tends to be larger in amplitude than the sum of the P3a responses to each of these deviances, indicating super-additivity for this ERP component. Beside the MMN component, equivalence of also the P3a and that of the RON in the ERPs to timely and earlier pitch deviances are tested in the present study to see if the individual P3a and RON responses to pitch and onset timing deviants interact when the deviances occur simultaneously and, if they do, to find out whether the interaction is sub-additive or super-additive.

The main research questions of the present study and the experiments in connection with these questions can be highlighted as follows: the first question addressed in the present study is whether the pre-attentive change detection mechanism benefits from regularity of stimuli. In other words, whether the pre-attentive change detection mechanism is entrained by isochronous stimuli in such a way that its sensitivity or responsiveness will be maximized at the most probable times of stimulus occurrence and minimized when this probability is relatively low. For this purpose, we compared the amplitudes of the MMN responses to timely and earlier stimuli, using the latter as a temporal probing stimulus delivered notably earlier than the standard onset timing. The second question we address is whether the temporal relationship among different components of the MMN differ when the occasional auditory change occurs earlier than the time hinted by the regular standard stimuli, which would suggest different processing of the timely and earlier-than-usual stimuli. We use the SCD maps of the MMN, P3a, and RON components of the electrical brain responses for this purpose.

## Materials and Methods

### Subjects

Ten healthy subjects (age range: 19–21 year; six females) with normal hearing participated in the present study. Healthy participants were fully informed about the study and gave written informed consent for their participation in accordance with procedures approved by the Human Research Ethics Committee of Koc University. During the recording sessions, participants were seated comfortably in a sound-attenuated and Faraday caged booth. They read a text of their own choice and were instructed to ignore the auditory stimulation.

### Stimulation

Stimulation paradigm was basically an odd-ball design with one standard and three deviants. Stimuli were sinusoidal tone pips of 50 ms duration with 10 ms rise and fall times. Standard tone-pips were presented with an IOI of 800 ms. Some of the standard stimuli (S) were occasionally replaced by a deviant stimulus which differed either in its pitch, or its onset timing (IOI = 500 ms), or in both, randomly. These deviant stimuli are denoted by TP (timely pitch-deviant), EO (early onset time-deviant), and EP (early pitch-deviant). The number of standards before a deviant was randomized between 4 and 8, so that the probability of a deviant was around 15%, which corresponded to a standard-to-deviant ratio of 6:1. The earlier-than-usual pitch-deviants (EP) were used as a probe stimuli to compare the MMN they elicit with the MMN elicited by timely pitch-deviant (TP). Standards (S) and earlier onset (EO) deviants served as control stimuli to obtain the reference ERPs to be used in MMN calculations.

Matlab running on a PC was used for designing the stimuli and their presentation in a pseudorandomized oddball sequence. The tone-pip stimuli obtained from the PC’s audio output were delivered at 60 dB (nHL) *via* an audio system with two loudspeakers. Five laboratory personnel, who were tested and found to have normal hearing, listened to the tone pip stimuli and the mean of their detection threshold levels was referred to as 0 dB (nHL) to express the sound intensities of stimuli in dB (nHL).

Experiments with one participant were conducted in eight blocks. Of the 315 stimuli delivered in each block, 270 were standards, 15 were timing deviants, 15 were timely pitch deviants, and 15 were early pitch deviants, so that the partial probability was around 5% for each of the three deviants. Consequently, the total number of stimuli was 8 × 315 = 2,520, of which 2,160 were standards, 120 were timing deviants, 120 were timely pitch deviants, and 120 were early pitch deviants. In four of the experimental blocks, 1,000 Hz and 1,200 Hz tone-pips served as standard and deviant stimuli, respectively, and in the other four blocks their roles were swapped. Block sequence was randomized within and among participants.

### EEG Recording

The subject wore a 21-electrode EEG cap (Electrocap, Ag/AgCl) including a fronto-central reference electrode at the centroid of the Fp1, Fp2, Fz triangle and an occipito-central ground electrode at the centroid of the O1, O2, Pz triangle. Two additional electrodes were attached to the earlobes. Electrode resistances were tested to be below 10 kΩ. Using a Micromed recording system (consisting of a SAM-32 amplifier and SystemPLUS Evolution software), the EEG filtered between 0.015–1,000 Hz and sampled at a rate of 4 kHz was continuously recorded throughout each session. One of the extra channels of the system was used to record the tone-pip signals simultaneously with EEG to accurately align the onset times of the pips for off-line averaging of the ERP epochs. We used an unusually high sampling rate of 4 kHz to maintain an adequately high sample rate for recording the sound stimuli. To obtain 120 sweeps for each deviant type, a typical experiment was conducted in eight consecutive 5 min-sessions separated by pauses between 1 and 3 min. Standard and deviant pitches were randomly swapped between sessions and an equal number of responses with opposite standard/deviant combinations of tones were collected.

### Data Analysis

The recorded EEG data were digitally filtered offline using a pass-band of 0.3–30 Hz and they were epoched to have 100 ms pre- and 500 ms post-stimulus intervals. Those epochs with amplitudes exceeding ±100 μV in any of the channels were discarded. The remaining epochs with each of the four types of stimuli were separately ensemble-averaged to obtain the responses R_S_, R_TP_, R_EO_, and R_EP_, denoting respectively the ERPs to standard, timely pitch-deviant, earlier onset-deviant, and early pitch-deviant stimuli. In order to achieve the maximal MMN amplitudes, the ERPs were re-referenced to averaged earlobe as the MMN is recorded with opposite polarities at electrodes above and below the level of the Sylvian fissure (Ritter et al., 1992). Grand average waveforms were obtained from the ERPs of the ten subjects who participated in the experiments.

DPs to timely (IOI = 800 ms) and early (IOI = 500 ms) pitch deviances were calculated from the ERPs as follows: “DP_TP_ = R_TP_ − R_S_” and “DP_EP_ = R_EP_ − R_EO_.” One of the reasons why R_EO_ (and not R_S_) was subtracted from R_EP_ is because a deviance presented too early in a sequence of repetitive tones with a constant IOI is known to already elicit a MMN (Ford and Hillyard, 1981; Nordby et al., 1988; Näätänen et al., 1993; Kisley et al., 2004); and we wanted to obtain the MMN to a pitch deviance alone, without a timing mismatch confound. The second reason why we chose the above procedure for derivation of the DPs was to avoid a possible confound due to an N1-effect which would associate with inadequate recovery of N1 just due to the decrease in IOI, regardless of whether the deviant is spectrally different from the standards preceding it. In this way, an inadequate recovery- or refractoriness-related N1 effect of this sort on both responses (R_EO_ and R_EP_) were balanced and canceled when one was subtracted from the other to obtain DP_EP_. Such an N1-effect imbalance was already not an issue when subtracting R_S_ from R_TP_ to obtain DP_TP_. Therefore, there may not be any difference, due to an IOI-related N1-effect, between the DP_EP_ and DP_TP_ waveforms. On the other hand, a pure MMN to an advance in onset timing cannot be obtained by subtracting R_S_ from R_EO_ because the N1 components of the two responses may not be equal, due to their different recovery states depending on different IOIs. However, because designing a standard stimulus that will have the shorter IOI and also the same stimulus history is problematic, if not impossible, the standard with an IOI different from the deviant IOI might be used as the standard for IOI-deviant for an approximate calculation of DP, as has been done previously in the literature (e.g., Paavilainen et al., 2001). Such an approximation is less inaccurate if the DP component to be studied is the P3a. This is because the P3a is far from the N1 in latency and, therefore, should be less affected by a possible N1-effect. Based on these arguments, we calculated the DP_EO_ = R_EO_ − R_S_ DP to obtain, at least approximately, the MMN and P3a responses to IOI-deviance to test the additivity of these responses in IOI and pitch deviances.

Because any systematic shifts in pre-stimulus potentials must have been canceled out due to the applied subtraction procedure, any non-zero pre-stimulus baseline of the difference potentials DP_TP_ and DP_EP_ must be the noise reflecting the EEG fragments that survive despite averaging. We did not prefer to measure the amplitudes with reference to these pre-stimulus baselines, because this would require superimposing the pre-stimulus noise on the noise already present in the post-stimulus latency range of interest, thus degrading the signal-to-noise ratio and increasing the variance in statistical analysis of the response wave amplitudes.

Amplitudes of the MMN, P3a, and RON deflections in individual DPs were measured as the mean amplitude over a 40 ms period centered around their peaks, which were defined as the negative maximum within 100–180 ms, the positive maximum within 200–300 ms, and the wide negative deflection within 350–450 ms latency intervals, respectively. Amplitudes of the late negative wave at a latency around 400 ms in individual potentials were measured, on the other hand, as the mean amplitude over the 350–450 ms latency range. Epoching, averaging, waveform subtraction, latency and mean amplitude reading, topographic plotting, scalp potential and current density (Laplacian) mapping were all carried out through using the MATLAB toolboxes EEGLAB (Delorme and Makeig, 2004) and ERPLAB (Lopez-Calderon and Luck, 2014).

One sample *t*-tests were used to see if the MMN and P3a components of the difference potentials DP_TP_ and DP_EP_ were significant deflections from the zero baseline. Significance of the mean amplitude difference between MMN_TP_ and MMN_EP_ were examined by means of a paired *t*-test because the two dependent variables were measured in the same session and from the same subjects. Using a freely available Web-based calculator (Rouder et al., 2009)^1^, we also applied a Bayes Factor approach to the MMN amplitude data. Since we had no a prior information about effect size and no expectation for a null hypothesis, we preferred the JZS previously suggested by Rouder et al. (2009) as the non-informative default. For the same reason, the scale of the prior on effect size, which may be tuned according to an expectation, was selected as 1; i.e., the default scale factor.

## Results

### Difference Potentials

**Figure 1:**
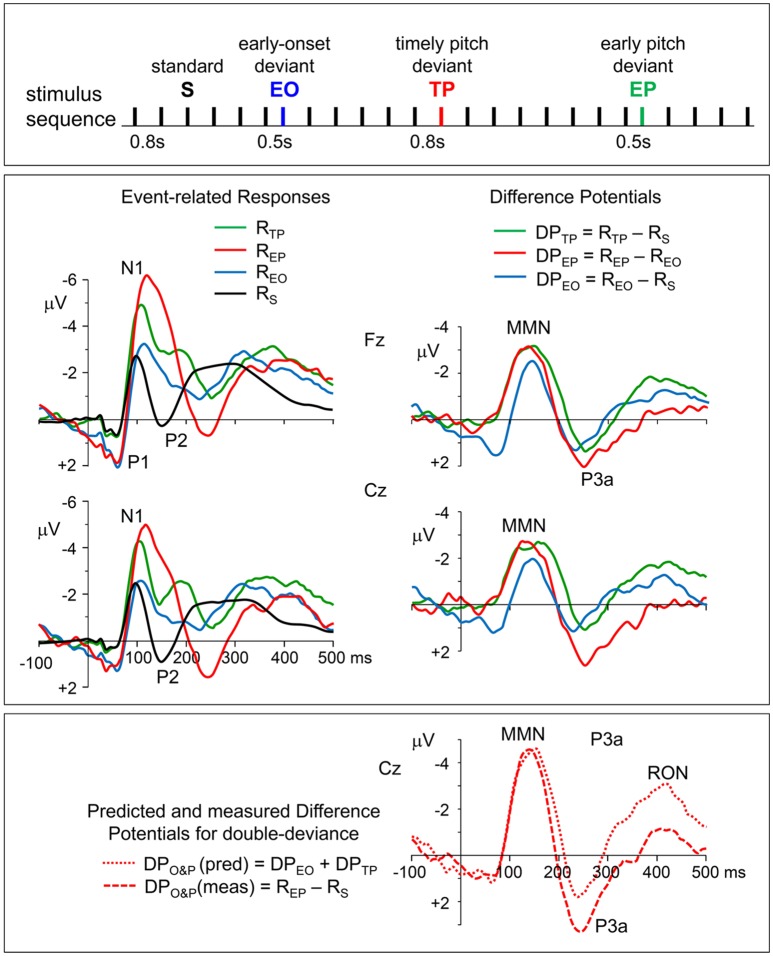
Top panel: stimulation paradigm. Standard inter-onset interval (IOI) between successive tone-pips was 800 ms. Deviant stimuli which differed from standard (S) ones either only in their pitch (TP), or only in their earlier onset time (EO), or in both (EP) were randomly distributed in the sequence in such a way that the number of standards before a deviant changed randomly between 4 and 8, maintaining a probability of around 5% for each of the three deviants. Middle panel, left column: grand average event-related potentials (ERPs) to timely pitch-deviant, early onset timing-deviant, earlier pitch-deviant, and standard stimuli (R_TP_, R_EO_, R_EP_, and R_S_, respectively). Re-referenced to averaged earlobes. Middle panel, right column: the difference potentials (DPs) obtained for timely (IOI = 800 ms) and earlier (IOI = 500 ms) pitch deviances and for onset deviance due to shortening of the IOI, which were calculated as DP_TP_ = R_TP_ − R_S_, DP_EP_ = R_EP_ − R_EO_, and DP_EO_ = R_EO_ − R_S_, respectively. Bottom panel: the two plots seen on the right of this panel with mismatch negativity (MMN), P3a, and reorienting negativity (RON) waves are the DPs calculated as DP_O&P_ (meas) = R_EP_ − R_S_ and DP_O&P_ (pred) = DP_EO_ + DP_TP_, to obtain, respectively, the actually measured DP to “IOI&pitch” double-deviant, and its model waveform predicted by assuming additivity of the responses to single deviants (onset or pitch).

In line with the general characteristics of auditory evoked potentials (Näätänen and Picton, 1987), all of the ERPs to the standard and deviant tone pips were recorded with maximal amplitudes from fronto-central electrodes Fz and Cz. Therefore, grand average waveforms of the ERPs recorded from these electrodes are presented in Figure 1 (left panel). Of the typical components of auditory ERPs (namely, P1 with a latency around 50 ms, N1 within a latency range of 90–120 ms, and P2 within a latency range of 140–200 ms), the first two are present explicitly in all of the four types of ERPs. The P2, however, is barely seen in the ERP to timing-deviant and it is obscured in the ERP to pitch&timing-deviant. A late negative wave, which may be identified with the RON (Schröger and Wolff, 1998), covers the 350–450 ms latency range in the ERPs to all three types of deviants.

Non-zero potential levels in the pre-stimulus period, which appear as baseline shifts in the ERP waveforms to early onset (EO) and early pitch (EP) stimuli, must be due to the fact that these relatively earlier stimuli occur before the responses to preceding stimulus have totally ended and reached to the zero baseline. However, because the difference potentials (DP_TP_ and DP_EP_) are derived from responses with identical stimulation history, such systematic pre-stimulus potentials must have been canceled out due to the applied subtraction procedure, and therefore, no systematic base-line shifts must have remained in these DPs. The fluctuations in the pre-stimulus periods of DP_TP_ and DP_EP_ should therefore be the noise reflecting the EEG fragments that survived despite averaging.

The ERPs of individuals, recorded from the midline frontal and central electrodes (Fz and Cz) with maximal amplitudes, were used in the calculation of DPs to obtain the MMN and P3a waves and in their statistical analysis. Grand average waveforms of these DPs are given in the right panel of Figure 1. The responses to timely pitch-deviant (R_TP_), to earlier onset timing-deviant (R_EO_), and to earlier pitch-deviant (R_EP_) stimuli are all clearly more negative than the response to standard stimulus (R_S_) in the latency range immediately following the N1, where an MMN is expected to occur. In fact, two one-sample *t*-tests conducted for the MMNs in Fz-recorded difference potentials DP_TP_ and DP_EP_ showed that the mean amplitudes ±20 ms around their peak latencies were significantly above zero baseline (respectively, *t*_(9)_ = 6.12, *p* = 0.0002 and *t*_(9)_ = 5.77, *p* = 0.0003), indicating that the responses to deviants were significantly greater in amplitude than the responses to their respective standards.

**Figure 2:**
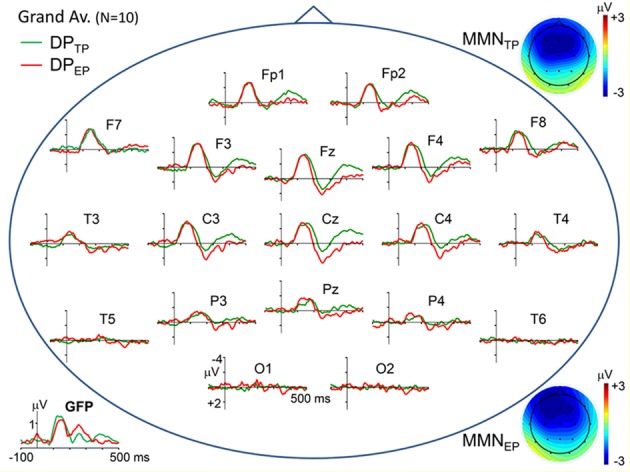
Scalp topographical plots of the grand average DPs to timely and earlier pitch deviances (DP_TP_ and DP_EP_, respectively). Potentials are re-referenced to averaged earlobes. Global field power plots of the grand average DPs are at the bottom-left corner. At the top-right and bottom-right corners, scalp potential maps of the MMN components (mean over 120–180 ms latency range) in the two DPs are given.

Topographic plots of the grand average “deviant-standard” DP waveforms, obtained for timely (IOI = 800 ms) and earlier (IOI = 500 ms) pitch deviances (DP_TP_ and DP_EP_, respectively) are given in Figure 2. In line with the typical characteristics of “deviant-standard” DP waveforms elicited in a MMN paradigm (Näätänen, 1990), the difference waveforms belonging to both types of stimuli consist mainly of a negativity called MMN at a somewhat longer latency than N1 and a positivity called P3a following the MMN. Because these difference waves have maximal amplitudes at electrodes Fz and Cz, the DPs at these electrodes calculated from the grand average ERPs to timely and early pitch deviances and to onset timing deviance are given in detail in the right panel of Figure 1. It is to be noted that only the DP to onset timing deviance (DP_EO_) has an early positive wave peaking at a latency of around 70 ms. Negative waves of all the three grand average DPs peaking at around 150 ms have similar maximal amplitudes of around 3 μV. Being the two DPs to be compared particularly, the MMN waves in grand averages DP_TP_ and DP_EP_ had very similar amplitudes slightly above 3 μV. Mean latencies of the Fz-recorded MMNs to timely and early pitch deviances and their standard deviations were 148 ± 16 ms and 146 ± 14 ms, respectively. Their mean amplitudes and standard deviations were −3.11 ± 1.26 μV and −3.16 ± 1.64 μV. The difference between the means of the MMN_TP_ and MMN_EP_ amplitudes was found to be non-significant (Fz: *t*_(9)_ = 0.157, *p* = 0.878). A Bayesian approach applied to the Fz-MMN amplitude data, to further quantify this null-result, gave a Bayes factor of 4.25, which is an odds ratio supporting the decision of accepting the null hypothesis.

Mean amplitudes and standard deviations of the P3a at electrode Fz for the timely and early pitch deviances were 1.60 ± 1.67 μV and 2.32 ± 1.68 μV, respectively. Two one sample *t*-tests conducted for the P3a deflections in Fz-recorded difference potentials DP_TP_ and DP_EP_ showed that the mean amplitudes ±20 ms around the peak latencies of these waves were significantly above zero baseline (respectively, *t*_(9)_ = 2.868, *p* = 0.019 and *t*_(9)_ = 4.151, *p* = 0.0025). Difference between these P3a mean amplitudes to timely and early pitch deviances proved to be significant (*t*_(9)_ = 3.156, *p* = 0.012).

The late component of the DPs, which appeared in the 350–450 ms latency range and was identified as RON, had maximal amplitudes at fronto-central electrodes (Figure 2). Mean amplitudes and standard deviations of this difference wave at electrode Fz for the timely and early pitch deviances were measured as −1.74 ± 1.01 μV and −0.39 ± 1.18 μV, respectively. Difference between these RON mean amplitudes to timely and early pitch deviances proved to be significant (*t*_(9)_ = 4.08, *p* = 0.003). However, in contrast to the DPs, the same wave appears to have similar amplitudes in the respective ERPs to timely and early pitch deviances given in Figure 1 (−2.71 ± 1.50 μV and −2.61 ± 1.40 μV, respectively), with no significant difference between them (*t*_(9)_ = 0.836, *p* = 0.423).

To check if the MMN and P3a responses to a change in IOI and to a change in pitch are additive when these changes occur simultaneously, the “double deviant—standard” DP actually recorded is compared with its predicted version, which is modeled as the sum of the two DPs obtained by subtracting the standard response from each of the responses to the two single deviants, assuming their additivity. The waveforms of the actually recorded and predicted DPs are given at the bottom of the right panel in Figure 1. Measured and predicted DPs to early pitch deviance (double-deviance of onset time and pitch) are calculated as follows: DP_O&P_ (meas) = R_EP_ − R_S_, and DP_O&P_ (pred) = DP_EO_ + DP_TP_. Concordance of the MMN amplitudes in the two waveforms indicates additivity of this ERP component. However, the measured P3a has a larger amplitude than the predicted one. This difference in favor of the measured P3a, which proves to be significant (*t*_(9)_ = 2.316, *p* = 0.046), not only disproves additivity, it further indicates super-additivity.

### Scalp Current Density (SCD) Maps

**Figure 3:**
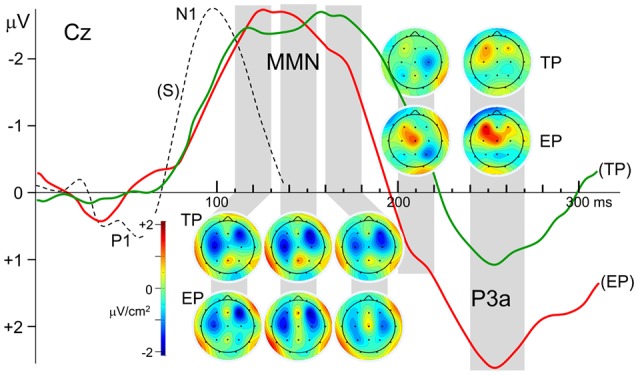
Laplacian maps displaying the scalp current density (SCD) distributions of the Grand average MMN and P3a components in the ERPs to timely and earlier pitch deviances (TP and EP, respectively). Maps were obtained for the mean Laplacians over the time intervals indicated by gray stripes. Please note that the current density color scale is the same for all maps. N1 deflection of the ERP to standard stimulus is given in broken lines for comparison of its latency with the latency of MMN.

Besides the very close amplitude similarity between MMN_TP_ and MMN_EP_ when they are evaluated as a single global negative wave within the latency range of 110–180 ms, there are some discrepancies between their waveforms, which become apparent when the central and parietal recordings given in Figure 2 are inspected in detail. The MMN waveforms in these scalp regions split into two subcomponents whose relative weights change depending on the recording site. Taking this multi-component feature of the MMNs into account, their SCD maps are computed for three successive latency ranges between 110–130 ms, 135–155 ms, and 160–180 ms, as illustrated in Figure 3. The two bilaterally located cortical current dipoles that an MMN should typically have are reflected in all these maps. In fact, the sinks of these dipoles are apparent, but the sources corresponding to these sinks, which normally take place around the mastoids with a nose reference, are seen at the lower sides only as faint positivity’s because of the averaged-ear reference. In the SCD maps of MMN_TP_ the initial balance between the current densities of bilateral sinks becomes impaired at later phases of the MMN in favor of the right one, and a slight midline frontal positivity (a superficial current source) emerges. In the SCD map of MMN_EP_, on the other hand, the sink at the right has a stronger current density than the sink at the left at all phases of the MMN starting from its beginning. This MMN also has a frontal source which seems to be coupled by a share from the right hemispheric sink to form a dipole. This dipole and the sink at the left gradually disappear at later times in contrast to the right-dominant sink pair of the MMN_TP_, which lasts up to the end of the whole MMN period. Furthermore, there seems to be a parietal sink in the SCD maps of both timely and early MMNs; the one for MMN_TP_, however, is stronger and more durable. The SCD distribution of the P3a component in the 240–270 ms latency range also displays a map with two bilaterally located dipoles. However, in contrast to the slightly right dominance seen in the dipoles of the MMNs, the left hemispheric dipole of the P3a is far stronger than the right one, especially in the case of early pitch change. A current source is evident already in the 200–220 ms latency range for the P3a to early pitch deviance but not for the P3 to timely deviance.

## Discussion

### Difference Potential Waveforms

#### Mismatch Negativity

The MMN, which is an estimate of the deviance effect, should be as free as possible from the influence of refractory effects or differences in stimulus characteristics that are unrelated to the deviance detection process itself. The issue that the standard and deviant stimuli in an oddball paradigm may evoke different cortical responses just because their frequencies are different was resolved in the present study by swapping the roles of the two frequencies in different blocks and averaging the respective ERPs. Another issue, which might stem from the difference between the times allowed for recovery of the responses to early deviants and timely standards, was also addressed and resolved by calculating D500 by subtracting RT (not RS) from RTP. In this way, the ERPs to be subtracted from each other were made to have identical stimulation histories, and the IOI-related N1 effects on both responses (RT and RTP) were balanced and canceled when one was subtracted from the other.

However, another refractoriness issue remains to be considered. It is related to a possible imbalance between the refractoriness of the N1 to standards which are repeated successively and frequently, and the refractoriness of the N1 to deviants which are presented singly and occasionally. However, a phenomenon called repetition suppression, resulting from successive presentation of identical auditory stimuli, has been shown to account for aspects of MMN generation (May et al., 1999), and is connected to predictive coding and trace formation (Baldeweg, 2007). A repetition positivity, which is an adaptation effect, develops in the standard ERP at 50–250 ms post-stimulus and largely contributes to the MMN within this wide latency range, including that of the N1. From this viewpoint, the relatively early part of the “deviant-standard” DP may not result only from an N1 refractoriness effect, and it probably bears a genuine MMN component. Therefore, controlling for the effect of a repeating standard may cause underestimation of the genuine MMN. Furthermore, in our study, two MMNs (those to timely and early deviances) are compared with each other. Both MMNs are equally affected from a possible N1 refractoriness imbalance of this sort between the respective responses, that are subtracted from each other to obtain the two DPs. Therefore, an N1 refractoriness effect may not be a critical issue for the present study, even if it existed.

The present MMN study with rhythmic standard auditory stimuli demonstrated that the MMN_EP_, elicited by deviants that are distinctly earlier than standard stimulus timing, are similar in their amplitude and gross scalp topography to the MMN_TP_ elicited by the deviants that occur at the times implied by rhythmic standard stimulation (see Figures 1, 2). The reason why we select the recordings made from the fronto-central electrode Fz for statistical evaluations of the MMN is because this ERP component is recorded with largest amplitudes, thus with best signal-to-noise ratio, from this fronto-central scalp areas. Assuming that the pre-attentive mismatch detection is indexed by MMN (Kujala and Näätänen, 2003; Näätänen et al., 2012), and that the mismatch responses to double-deviances in the physical features of a stimulus, including its onset timing, are additive (Levänen et al., 1993; Schröger, 1995; Takegata et al., 1999; Paavilainen et al., 2001), the similarity between amplitudes of the MMN_TP_ and MMN_EP_ in the present study speaks against a stimulus-driven modulation of the pre-attentive change detection mechanism by rhythmic stimuli, or a possible automatic mismatch detection advantage involving some involuntary or covert temporal expectancies. However, there are studies in the literature providing evidence for smaller MMN amplitudes with irregular stimulus sequences than with rhythmic ones (Imada et al., 1993; Takegata and Morotomi, 1999). These findings may indicate a change detection mechanism whose sensitivity is modulated by the current probability of stimulus occurrence. However, as explained below, the objectives and methodology of these studies are not quite compatible with the particular question treated in the present study.

In the work of Takegata and Morotomi (1999), subjects were presented with sequences of tones delivered at two IOIs. Each sequence included either one or both of the IOIs. In sequences with two IOIs, the order of the IOIs was alternated or sequenced randomly. The amplitude of the MMN they recorded was larger in sequences with one IOI than in those with two. However, alternation or randomization of IOI may have an adverse effect on formation of the memory trace of standard stimulus (Winkler et al., 2001), and thus, may result in a reduction in MMN amplitude. Such a reduction would therefore be due to loss of the temporal regularity advantage in the trace formation phase and not due to a possible drop in the performance of the change detection process involving its comparison phase. It may not indicate, therefore, a modulating effect of rhythmic stimuli on the responsiveness of the change detection mechanism.

In the mismatch field (MMF) study of Imada et al. (1993), the effect of several parameters on the amplitude of the MMNm (the magnetic counterpart of the MMN) was investigated. When a constant inter-deviant interval was employed and the number of standards between two deviants varied in different sessions, MMF amplitude increased as the number of standards increased, indicating a positive effect of decreased probability of deviant stimuli on the amplitude of the MMNm. However, this positive effect disappeared when ISI in a sequence was made to vary widely. They concluded, therefore, that increasing the number of standards between two successive deviants reinforces the trace, and the lack of stimulus regularity reduces the reinforcing effect of increasing the number of standards. They further concluded that the timing of the stimulus sequence is preserved in sensory memory in addition to the physical features of the stimulus, implying an interaction between processing of sequential and physical features of the stimulus. This would mean non-additivity of the responses to mismatches in sequential and physical stimulus features, suggesting that the mismatch response to a change in the timing or a physical feature (e.g., pitch) of the stimulus will be smaller in amplitude when both changes occur simultaneously rather than when either of them occur individually. However, because they provided the normalized values of the MMFs recorded in three conditions, no comparison can be made to directly see if regularity of stimulus presentation actually caused an increase in the MMF amplitude.

In the relatively recent study of Schwartze et al. (2011), on the other hand, no significant difference was found between the MMNs that were recorded with isochronous and random stimulus sequences. Such a finding might have been taken as evidence supporting the viewpoint that temporal regularity of stimuli does not provide an advantage for the mechanism automatically detecting a change in stimulus features. However, even if such a change detection advantage existed, it might not have been reflected in their results as superiority of isochronous stimuli over random stimulation, in terms of MMN amplitude. This is because such an expectation-based advantage is for the nearly on-time and even delayed deviants, whereas the negative effect of temporal irregularity on MMN will be specific to deviances for the stimuli occurring distinctly earlier than expected. This argument is not related to a possible refractoriness of the system for an early presentation of stimuli, but to the possibility that the change detection mechanism whose sensitivity is presumed to follow the time course of stimulus occurrence probability; i.e., the possibility that it is entrained to the rhythm of the regular stimuli and may not be fully sensitive to detect the mismatch in a stimulus earlier than implied by the average IOI. We mean a sort of idling during time periods of low stimulus probability, not refractoriness of the system. Of the stimuli employed in the mentioned study, only those with IOIs between 500 ms and ca. 700 ms (about 30% of all the stimuli) would fall into the category of “distinctly earlier,” considering the 900 ms average IOI in their experiment. Therefore, an isochronous vs. random MMN difference might have failed to reach statistical significance, even if it existed. In a later study of the same group (Schwartze et al., 2013) no significant interaction was found between timing- and pitch-deviant responses even when the analysis was restricted to the IOI range between 400 ms and 800 ms (about 50% of all the stimuli). However, the responses evaluated in that study were P1 and N1 and not MMN.

Another confound which might have masked, in the mentioned study, a possible mismatch detection advantage provided by temporal regularity would be the following: the expectation to record smaller amplitude MMN with random IOI is based on the hypothesis that the advantage, presumably provided by temporal regularity and utilized by the mismatch detection mechanism, will be lost when IOI is randomized. However, with random IOI, the detector mechanism would need to be continuously functional because there is no specific or “expected” time at which the next stimulus is likely to occur. Therefore, the mechanism will constantly be as responsive as it would be around the expected stimulus arrival times in an isochronous sequence. This may be an alternative reason why a possible MMN advantage of the isochronous sequence might have been masked in the study of Schwartze et al. (2011). These two possible confounds have been circumvented in the present study because we have directly tested if the MMN elicited by deviants distinctly earlier than the usual timing of stimuli is different in amplitude compared to the MMN elicited by the deviants occurring on-time, i.e., at instants hinted by standard timing.

Although their main interest was testing the hypothesis of additivity of the MMNs elicited simultaneously by more than one deviant, Paavilainen et al. (2001) also recorded the MMN to a pitch deviance occurring earlier than it was implied by the SOA. They compared the actual-recorded MMN to an earlier pitch deviance (their “fre&SOA”), with its expected waveform modeled as the sum of the individual MMNs to pitch (their “fre”) and timing (SOA) deviances. However, in the calculation of the MMN to SOA deviance, they subtracted the ERP to standard stimulus from the ERP to SOA deviance. Because the stimulation histories of these two ERPs were not identical, the difference between their exogenous parts might show up as an N1 effect in the DP. Furthermore, in the calculation of their modeled double-deviant MMN, they used the MMN elicited by pitch deviances occurring timely (i.e., at instances cued by rhythmic standards), instead of an unknown pitch-alone MMN that would be elicited by a pitch deviance occurring earlier than that. In other words, they assumed that this hypothetical MMN was identical to the MMN to timely (i.e., with long pre-SOA) pitch deviance, which might not have been the case because the sensory memory trace of the standard may be less attenuated for the earlier deviant (Kaukoranta et al., 1989; Näätänen et al., 2007). However, it was shown in the MMF study of Imada et al. (1993) that, although MMF decreased slightly when the interstimulus interval just preceding the deviant (their pISI) increased from 0.6 to 3.4 s, decay of the memory trace was negligible during this wide range of ISIs. Furthermore, the results of the present study indicating the similarity of the MMNs to timely and earlier deviances also support the assumption of Paavilainen et al. (2001), that the MMNs elicited with pre-deviant IOIs of 500 ms and 350 ms in their study were identical.

Above, we discussed previous studies addressing MMN in rhythmicity contexts. They have focused, however, on research questions such as whether temporal sound structure is processed independently from other sound attributes or whether the MMNs elicited simultaneously by more than one deviant are additive. We have also addressed MMN in rhythmicity contexts in the present study, but to answer the question as to whether the auditory change detection process below attentive levels is modulated by or entrained to the rhythm of an isochronous auditory stimulation, pre-attentive facilitation of involuntary temporal extrapolations and expectancies might have been speculated. Our electrophysiological results, however, do not support a benefit of regularity-cued timing of the stimulus for the pre-attentive mechanisms of change detection. Therefore, pre-attentive mechanisms may not be contributing to the advantage of more accurately judging rhythmically expected tones, as mentioned in the study of Jones et al. (2002). The source of such an advantage seems to be an outcome of the higher cognitive processes involving consciousness and attention.

Insensitivity of MMN to predictability of the occurrence of deviant stimuli was previously demonstrated by Scherg et al. (1989). In their regular condition the deviant stimuli were presented systematically after a certain number of standards. In their irregular condition the deviant stimuli were presented at the same probability but were randomly dispersed among the standard stimuli. They found no difference in the amplitude or latency of the MMNs obtained in the two cases. However, in their study, there was some uncertainty in the order of the deviant in a sequence of stimuli separated by equal inter-stimulus intervals. Subjects did not have a cue indicating if the next stimulus will be a standard or a deviant, but onset time of the next stimulus was exactly predictable. In the present study, on the other hand, we compared the MMNs to deviant stimuli with predictable and unpredictable stimulus onset timing and found out that they are similar, indicating a different kind of MMN insensitivity to predictability. In our case, the uncertainty is in the timing of deviant stimulus (i.e., “when” is the issue) and not in its position in a regular stimulus sequence as in the work of Scherg et al. (1989), where “which” was the issue.

At this point, we should mention a possible weakness of the present study. The issue is related to the choice of standard and deviant stimulus frequencies. Some studies suggest that MMN amplitude actually reflects the magnitude of discriminability and not the magnitude of difference (e.g., Horváth et al., 2008a). The frequency difference between the standard and deviant stimuli in the present study (i.e., 1,000 vs. 1,200 Hz difference) is a readily detectable one. Thus, the observed lack of a timeliness advantage may have been caused by a plateau-effect; i.e., the hypothetical timeliness advantage, that early deviances lack, may not have been sufficient to meaningfully reduce discriminability. Testing whether our results were susceptible to such a plateau effect requires a series of further experiments in which deviant stimuli with various discriminability levels starting from a threshold of, for instance, 8 Hz difference (Sams et al., 1985), may be employed.

#### P3a Component

Despite the similarity of amplitudes of the MMNs to timely and earlier pitch deviances in the present study, the amplitude of the consecutive wave called P3a appeared to be significantly larger for a pitch deviance that was earlier than its regular timing. Typically, the P3a is elicited in response to occasional “distractor” stimuli when the subject is actively attending to target stimuli (e.g., of a different pitch) embedded in a stream of frequently presented standard stimuli (Squires et al., 1975; Näätänen, 1990, 1992; Katayama and Polich, 1998; Dien et al., 2004; Polich and Criado, 2006; Rinne et al., 2006). However, in a typical MMN recording where there is no target stimuli the subject has to actively attend to, this positive response component can still be assumed to represent the involuntary capture of attention (Friedman et al., 2001) by the deviant stimuli, which is in fact directed to a task such as reading a book. The same argument may apply in the present study where pitch, timing, and pitch&timing deviants acted as distractor stimuli, all eliciting the P3a response to be associated with involuntary attention shift *via* a stimulus-driven bottom-up process, as mentioned by Escera et al. (2000). The finding of an enhanced P3a response to a deviance earlier than its usual timing may indicate that such a timing-deviant stimulus more effectively triggers the call-for-attention mechanism suggested by Öhman (1979) and the following attention switching (Escera et al., 2000), which results in orienting passive or spontaneous attention described by James (1890). Our results show that the P3a to an early pitch change is not only larger in amplitude than the P3a to a single pitch or an onset timing deviance, it is even significantly larger than the sum of the P3a responses to these single deviants. A similar observation is reported, though without statistical support, in the work of Takegata et al. (1999), where the interaction of responses to stimulus feature (locus of origin) and conjunction deviants are studied. Paavilainen et al. (2001) also observed super-additivity of the P3a responses, only in the DPs to double-deviances in the frequency and inter-onset asynchrony of stimulus, and provided statistical support for significant difference between the recorded response and its model based on additivity assumption. The super-additivity which occurs in the present study when a pitch deviance is accompanied by a simultaneous shortening of the IOI is probably related to an increased sensitivity of the neural circuit for stimulus-driven automatic capture of attention.

#### RON Component

The later negative activity around 400 ms, which may be identified with RON (Schröger and Wolff, 1998), is seen in the DP to timely pitch deviances but not in the DP elicited by earlier pitch deviances. This component has been shown to provide a neurophysiological index of reorienting of attention which has been automatically switched to the most recent deviant stimulus (Berti et al., 2004). The RON is usually measured when auditory stimuli are attended and it may not even be elicited when the sounds are ignored. Recording of a RON in the present study, where the participants were instructed to ignore the sounds, might therefore seem to be an unexpected finding. However, recording a clear P3a indicates an involuntary capture of attention by the deviants even when they are not task-relevant. Recording of a RON in the present study should not therefore be considered as surprising.

Missing of a RON in the averaged waveform of the DP to earlier pitch deviance, despite an enhanced P3a, is not, in fact, due to its absence in the case of an early deviance because this wave is certainly present in the recorded response R_EP_ (see Figure 1). The reason why it does not appear in the DP to early pitch deviance (DP_EP_) is because the RON in the ERP to that deviance is similar in amplitude to the RON in the ERP to IOI-deviance, and they cancel each other when the latter is subtracted from the former to obtain DP_EP_. In other words, apparent absence of this late negative wave in DP_EP_ is due to highly sub-additive behavior of the RON, which is clearly demonstrated in Figure 1 (bottom-right waveforms) where the RONs in predicted and recorded DPs to “IOI&pitch” double deviant are compared. On the other hand, in the case of DP to timely pitch deviance (DP_TP_), the subtracted ERP is that to standard stimulus which elicits no RON, and the RON in the ERP to timely pitch deviance is directly reflected in the difference potential DP_TP_. The same would occur and a RON would also appear in the DP for early pitch deviance if the response to standard (R_S_) is subtracted from R_EP_. In fact, this can be done flawlessly for evaluating rather late responses like RON, because at such long latencies the N1-effect should not be an issue and R_S_ may directly be used for subtraction. Therefore, it can be concluded that the reorienting of attention that has been switched to an earlier-than-usual deviance is not stronger than that switched to a timely deviance, although the former causes more effective attention switching than the latter, as indicated by a significantly enhanced P3a for the former.

### SCD Maps

#### Mismatch Negativity

Within the limitations of rather low spatial resolution provided by 19 channel EEG, the current sources and sinks corresponding to the right frontal and bilaterally temporal components of the MMN are reflected in the SCD maps given in Figure 3. It is also possible to roughly follow the variations in the strength of the components in different phases of the MMN and the dipolar processes, which occur at later latency ranges. Interestingly, the CSD map of MMN to early pitch deviance (EP) reveals a frontal dipole at latencies as early as 110–130 ms with its sink overlapping the right temporal sink of the auditory cortical MMN dipole. It is probably because of this overlap that the right hemispheric MMN sink appears to be more frontally located and stronger than the left hemispheric one, as was also observed in the MMN equivalent current dipole results reported by Alho et al. (1998) in their MEG study. The early appearance of the frontal MMN is rather surprising because, according to some studies, this MMN component is expected to lag the temporal MMN (Opitz et al., 2002; Doeller et al., 2003). An explanation for this early frontal activity observed in the present study may be as follows: although the P1 deflections with 70 ms latency in the ERPs to “early pitch” and “early onset” stimuli (see Figure 1) are canceled due to R_EP_ − R_EO_ subtraction, and therefore, no deflection peaking earlier than 120 ms appears in the difference potential (DP_EP_), an early effect of the IOI-deviance on processing of pitch deviance may continue to exist on the presumed attentional call mechanism and the following attention switching processes. In fact, deviance-related modulations of the middle-latency responses (MLR) demonstrated by Althen et al. (2016) suggest thalamic or primary cortical involvements which may cause P1–related early effects on later processing of change detection.

There are other studies indicating deviance processing earlier than MMN in human and animal brains. In Grimm and Escera (2012), one can find a review of such studies indicating adaptive MLR to repetitive auditory stimulation (Boutros and Belger, 1999; Müller et al., 2001) and reporting enhanced MLR components to deviant compared to standard sounds (Sonnadara et al., 2006; Slabu et al., 2010; Grimm et al., 2011, 2016). Results of an MMN study using SCD mapping (Yago et al., 2001) also support a multiple level processing of auditory deviance with sub-cortical involvement. The authors reported that the deviance-related significant density increase began at 94 ms and 154 ms over right frontal and temporal areas, respectively. They suggested two explanations for this early activity at right frontal areas. Their first explanation was based on a refractoriness-related N1 difference. This explanation, however, seems unlikely in our case because the earliest part of the MMN_EP_ deflection, peaking at around 120 ms, is fairly far away from the N1 peaking at around 97 ms, as can be seen in Figure 3. This latency difference of more than 20 ms indicates that the MMN DP may not have resulted simply from an N1 enhancement, due to deviant-activated fresh cortical units that have not been adapted to the frequency of the standard stimuli, like those units that are sensitive to that frequency. The second explanation the authors gave was that the early frontal activation may correspond to a genuine frontal MMN component, which may be fed earlier than the supratemporal cortex *via* the thalamic contribution to MMN generation. This latter explanation involving a subcortical contribution may also apply to our observation that the frontal SCD dipole is already present in the earliest phase of MMN_EP_. A larger amplitude of the P3a in the DP to early pitch change, compared to that in the DP to timely change, may also be considered as a continuing effect of this expedited processing. The earlier drop of the MMN_EP_ deflection compared to MMN_TP_ may also be due to advancing of the frontal component in time, in the case of early pitch change.

Rinne et al. (2000) reported in their EEG and MEG study that the frontal MMN generators to duration deviant tones are activated slightly (on average by 8 ms) after the supratemporal auditory cortex. Temporal priority of the auditory cortical component over the frontal one was also reported by Opitz et al. (2002). In their fMRI/ERP study, the strengths of the temporal and frontal activations are correlated with the amplitude of the change-related ERP at around 110 ms and 150 ms, respectively. In another fMRI/ERP study (Doeller et al., 2003), the behaviors of the early (90–120 ms) and late (140–170 ms) phases of the MMN were found to be parallel to the behaviors of the right superior temporal and right inferior frontal gyri, respectively. In a later study of Shalgi and Deouell (2007), however, involvement of a parallel, distributed processing type of network, rather than a canonical one, was suggested for MMN generation, based on their finding that the frontal component survived in their experiments, despite abolishment of the temporal component.

In all of the studies mentioned above, the change in single-deviants was in the duration or frequency of the stimulus as in the case of our timely pitch deviants without a simultaneous IOI-change. It is seen in Figure 3 that the CSD maps of the MMN_TP_ have a frontal component throughout the whole period of MMN covering the latency range of 120–170 ms. And the source of the frontal SCD dipole tend to get stronger at the last MMN period of 160–180 ms, in agreement with the dynamics of this component described in the mentioned studies. The bilateral SCD sinks of the temporal MMN component accompanies this frontal dipole with its sink overlapping the sink of the right temporal dipole. In the last MMN phase, the temporal component loses its strength so that the frontal dipole becomes more prominent. In the MMN to early pitch deviance (MMN_EP_), on the other hand, the frontal dipole is stronger in the earlier phases and it almost disappears in the last MMN period, which is probably due to the effect of simultaneous IOI-deviance with thalamic and/or primary cortical involvement, as mentioned above. For the attenuation of frontal component in later periods of MMN_EP_, there might be an alternative explanation based on the sub-additive behavior of this component for double deviants (Wolff and Schröger, 2001; Paavilainen et al., 2003). In the calculation of MMN_EP_ in the present study, the response to IOI deviance is subtracted from the response to IOI & pitch deviance, assuming additivity of the MMN responses to individual IOI and pitch deviances with no interaction between them. This may not be an issue for temporal components of the MMN, which is widely believed to be additive, but may cause an amplitude reduction in its frontal component which displays sub-additivity. This might be another reason why the late component of the MMN and the SCD sink corresponding to that component are attenuated in MMN_EP_ (see Figure 3).

SCD signature of the frontal MMN component appears as a source/sink combination corresponding to a dipole (see the maps in Figure 3, especially those of the MMN_EP_), similarly to the SCD maps obtained from 128-channel high-resolution ERP recordings in the study of Kasai et al. (1999). Their MMN, however, has a latency of around 200 ms which is somewhat longer than usual. This is probably because their ERPs were recorded during a selective attention task, and this might have caused overlapping of MMN with a relatively large N2b wave with a typical latency of 200 ms (Näätänen, 1992).

In our results, MMN_EP_ seems to have a more prominent frontal current source than MMN_TP_. This amplitude primacy of frontal MMN_EP_ should indicate a stronger call-for-attention towards a change in stimulus feature (pitch) when this change occurs earlier than the usual time of stimulus onset, considering the established views linking the frontal MMN component to that function. This viewpoint is also in harmony with our finding that the amplitude of the P3a to an early pitch deviance is larger than that to a timely deviance, because the P3a is associated with attention switching upon a call released by the frontal mechanism of MMN (e.g., Escera et al., 1998; Schröger and Wolff, 1998).

Both MMNs to timely and early pitch deviances have a parietal current source in their early phase, simultaneously with the temporal and frontal dipoles. In the map of MMN_TP_, this source reaches a maximum strength at around 150 ms and starts diminishing around 170 ms. The parietal source in the map of MMN_EP_, which is already weak initially, starts diminishing further at an earlier latency of around 150 ms. The observation that the parietal component shows up already in the initial phase of the MMN agrees with the observation of Kasai et al. (1999): the parietal dipole in their high-resolution ERP recordings starts to be seen in the earliest SCD map provided for 160 ms. However, this component continues to exist in their results, even with increased prominence, in the maps for 200 ms and 240 ms. The latter two latencies correspond, however, to the time ranges in which the MMN in the present work had already diminished and a prominent P3a emerged. As has also been argued above, this latency discrepancy may be because their ERPs were recorded during a selective attention task, causing interference of an N2b wave with the MMN. Based on their observation that parietal sources to various deviants did not differ significantly in location, Levänen et al. (1996) speculated that this MMN component might reflect the activity of more global and nonspecific change detectors, probably reflecting the activation of the polysensory cortex.

In summary, dynamics of the recorded MMN waveforms and their SCD maps indicate that the temporal, frontal, and parietal MMN components are simultaneously present within a certain post-deviance time window, rather than displaying a sequential appearance in time. This simultaneity suggests a parallel deviance processing in the supra-temporal, right frontal, and parietal cortices, all initiated by signals from deviance detection circuits in the thalamus and primary areas of the auditory cortex. As generally assumed (Näätänen, 1992), the frontal MMN component is probably related to call-for-attention, which is believed to cause an involuntary attention shift reflected by the later P3a wave, whereas the temporal MMN component is concerned with further feature-specific processing of the deviant stimulus. Besides this modality–specific processing of deviant, parietal component is assumed to be concerned with common (integrated) processing of possible changes in different stimulus features (Levänen et al., 1996). The results of the present study suggest that all of these processes speed up if the change is accompanied by an advance in regular stimulus timing, i.e., when the change occurs earlier than usual.

#### P3a Component

The SCD distribution of the P3a response to timely pitch deviance (TP) is basically a map with two bilaterally located sources within the latency range of 240–270 ms (Figure 3). Left-dominance of the bilateral sources observed is in agreement with the MEG results of Alho et al. (1998), who reported that a left hemisphere equivalent current dipole could be modeled for the magnetic counterpart of the P3a in seven participants, and a corresponding right hemisphere dipole could be modeled only in three participants. They also found that the average dipole moment was somewhat higher for the left hemisphere than for the right hemisphere.

A map of the P3a to early pitch deviance (EP), on the other hand, reveals a dominating frontal and a relatively weaker central source that are fused into an obliquely oriented elongated one. This duality of the P3a sources is in agreement with the two-component structure of this potential, consisting of an early midline-central and a later frontal sub-component (for a review, see Escera et al., 1998). Indeed, in the SCD map for about 40 ms earlier latency range (200–220 ms), this time the mid-line central member of this compound current source appears to be the dominating one. However, interestingly, in the SCD map of the P3a to timely pitch deviance there is not a prominent current source in this relatively shorter P3a latency range; only the remnants of the decaying right temporal MMN dipole can be seen. This difference between the P3a responses to timely and early deviances indicates a faster attention switching towards the earlier-than-usual pitch deviance. Furthermore, the finding that the frontal P3a source to the early deviance is much stronger than that to the timely deviance speaks for a stronger attention switching effect of the early deviance. Despite the interhemispheric asymmetry of the P3a in magnetic recordings that has been mentioned above, the electrically recorded novelty P3a was reported to be bilaterally symmetrical (e.g., Friedman and Simpson, 1994; Knight, 1996). In contrast to these earlier observations, the left fronto-central dipole of the P3a in the present study is much stronger than the right one, especially in the case of early pitch deviance. This may be because an IOI-deviant has not been used in any of the studies concerning the scalp topography of this ERP component. We may speculate, therefore, that this atypical P3a topography would be because the pitch deviance is coupled in the present study with a simultaneous IOI-deviance, rather than a deviance in another auditory feature of the stimulus such as its intensity, duration, or lateralization. In other words, this atypical observation may be due to the interaction which takes place between the P3a-related neural circuits, triggered by pitch-deviance and IOI-deviance when the two events occur simultaneously, forming a double-deviant. Such an explanation seems likely considering the super-additive behavior of the P3a in such interactions as demonstrated in the present study and also in Paavilainen et al. (2001).

Although it has been possible to reveal the basic SCD components of the MMN by employing a 21-channel recording system in the present study, we should admit that some finer but significant differences between the SCD maps of the MMN_TP_ and MMN_EP_ have probably been obscured due to the relatively low spatial resolution that could be achieved by this system. A spatio-temporal component analysis of the MMN waveforms recorded with better spatial resolution would have provided the potential to investigate further and more accurately determine the cortical sources and associations of the frontal dipole, as well as the parietal SCD-source observed in the present study for a deviance earlier than its usual timing. Especially the right frontal dipole, revealed in the SCD maps with an orientation tangential to the scalp, seems to be an interesting finding to be further investigated by means of magnetoencephalography, which is particularly sensitive to tangentially oriented dipolar sources in the cortex.

## Conclusion

The main findings of the present study, investigating how the auditory pre-attentive processing of stimulus-change and the subsequent involuntary attentional processes are affected from occasional shortening of the otherwise fixed interstimulus interval, can be summarized as follows, together with their implications:

1. The MMN to timely and early pitch deviances were recorded with similar amplitudes, indicating that isochronous stimulation does not have a modulating effect on pre-attentive change detection. We conclude, therefore, that the regularity of stimulus timing does not provide any benefit for the pre-attentive mechanisms of auditory change detection.
2. Right frontal MMN was revealed in the SCD maps as a superficial dipole suggesting a tangential orientation. This SCD dipole of the MMN to an early deviance was notably stronger and faster than that to a timely deviance, suggesting that an auditory change in a stimulus occurring earlier-than-usual initiates a faster and more effective call for attention.
3. A pitch-deviant auditory stimulus occasionally presented with a shorter IOI elicited an enhanced P3a. Furthermore, the P3a elicited by such a “pitch & IOI” double-deviant displayed super-additivity, suggesting a stronger attention switching to a change occurring in an earlier-than-usual auditory stimulus.
4. The late negative wave around 400 ms, which is associated with reorienting of attention, had similar amplitudes in the ERPs to single (pitch only) and double (pitch & IOI) deviances due to sub-additivity of this wave. This finding suggests that the reorienting of attention that has been switched to an earlier-than-usual deviance is of the same strength with that switched to a timely deviance, although a more effective attention switching to the former deviance was indicated by a significantly enhanced P3a to that deviance.
5. Dynamics of the recorded MMN waveforms and their SCD maps indicated that the temporal, frontal, and parietal MMN components are simultaneously present rather than emerging sequentially in time, suggesting a parallel deviance processing in the supra-temporal, right frontal, and parietal cortices, probably all initiated by signals from deviance detection circuits in the thalamus and primary areas of the auditory cortex.

## Author’s Note

Preliminary findings of this work were presented in MMN 2015—7th Mismatch Negativity Conference, 8–11 September 2015, Leipzig, Germany.

## Data Availability

The data supporting the conclusions of this manuscript will be made available by the authors, without undue reservation, to any qualified researcher.

## Author Contributions

PU conceived and designed the study, supervised the experiments, and wrote the manuscript. HK and SY prepared the experimental set-up and software for stimulus presentation and EEG recording, processed the data, did the statistical analyses, and contributed to manuscript revision. PU and HK conducted the experiments. All authors (except SY, who passed away before submission), read and approved the submitted version.

## Conflict of Interest Statement

The authors declare that the research was conducted in the absence of any commercial or financial relationships that could be construed as a potential conflict of interest.

## Footnotes

^1^http://pcl.missouri.edu/bayesfactor

## Abbreviations

DP: Difference Potential
DP_EP_: Difference Potential to early pitch deviance
DP_TP_: Difference Potential to timely pitch deviance
ERP: Event-related Potential
IOI: Inter-onset Interval
MMN: Mismatch Negativity
MMN_EP_: Mismatch Negativity to early pitch deviance
MMN_TP_: Mismatch Negativity to timely pitch deviance
P3a: The positive component around 250 ms of the ERP to deviant stimulus
R_EP_: ERP to early pitch deviant stimulus
R_EO_: ERP to standard stimulus with early onset
R_TP_: ERP to timely pitch deviant stimulus
R_S_: ERP to timely standard stimulus
RON: Reorienting negativity
SCD: Scalp Current Density.
